# ORC1 expression features and its prognostic significance in HBV-associated hepatocellular carcinoma

**DOI:** 10.3389/fonc.2026.1938736

**Published:** 2026-10-26

**Authors:** Rong Xie, Qing-Mei Li, Hang Wang, Tu-Mei Su, Qian-Bing Yin, Long Huang, Hong-Qian Liang, Yan-Fei Feng, Zi-Li Lv, Qian Wang, Ming-Hua Su, Bo-Bin Hu, Jian-Ning Jiang

**Affiliations:** 1 Department of Infectious Diseases, The First Affiliated Hospital of Guangxi Medical University, Nanning, Guangxi, China; 2 Department of Pathology, The First Affiliated Hospital of Guangxi Medical University, Nanning, Guangxi, China; 3 Key Laboratory of Early Prevention and Treatment for Regional High Frequency Tumor(Guangxi Medical University), Ministry of Education, Nanning, Guangxi, China; 4 Guangxi Key Laboratory of Early Prevention and Treatment for Regional High Frequency Tumor, Nanning, Guangxi, China

**Keywords:** biomarker, early-stage HCC, HBV, HBV-HCC, ORC1, prognosis

## Abstract

**Objective:**

Hepatitis B virus (HBV) infection is a leading cause of hepatocellular carcinoma (HCC). Conventional biomarkers alpha-fetoprotein (AFP) and des-gamma-carboxy prothrombin (DCP) have diagnostic limitations, particularly for early-stage disease. This study investigated the expression profile of ORC1 in HBV-related liver disease and its association with HBV infection, disease progression, and prognosis.

**Methods:**

178 participants were recruited: 124 HBV-positive cases (49 HBV-HCC, 33 HBV-cirrhosis, 42 chronic hepatitis B) and 54 HBV-negative controls (35 non-HBV-HCC, 19 hepatic hemangioma). Tissue ORC1 was detected by immunohistochemistry, and serum ORC1 was measured by ELISA in a subset of 17 HBV-HCC patients with available serum. Logistic regression identified factors associated with high ORC1 expression. ROC curves were used to evaluate the discrimination of tissue ORC1, AFP, and DCP for HBV-HCC, and the correlation between ORC1 and tumor diameter was analyzed. TCGA-LIHC data were used for external validation.

**Results:**

Tissue ORC1 was significantly upregulated in HBV-positive patients, and HBV infection was independently associated with high ORC1 expression (P<0. 001). ORC1 expression increased progressively from chronic hepatitis B to cirrhosis to HCC. In a subset of 17 HBV-HCC patients with available serum, tissue and serum ORC1 were moderately correlated (Spearman rs=0. 554, 95% CI 0. 085–0. 822, *P* = 0. 021). Tissue ORC1 showed moderate discrimination between HBV-HCC and HBV-LC (AUC = 0. 64) and between HBV-HCC and CHB (AUC = 0. 66). ORC1 expression was not significantly associated with tumor diameter (*P*>0. 05). High ORC1 expression was associated with poorer overall survival in TCGA-LIHC (HR = 1. 7, *P* = 0. 0026).

**Conclusion:**

ORC1 upregulation is independently associated with HBV infection and increases with disease progression in HBV-related liver disease. Although tissue ORC1 showed only moderate discrimination for HBV-HCC, high ORC1 expression was associated with poorer overall survival in TCGA-LIHC. These findings suggest that ORC1 is primarily a proliferation- and progression-related marker in HBV-related liver disease.

## 1 Introduction

Primary hepatocellular carcinoma (HCC) exhibits strong malignant invasiveness. According to GLOBOCAN 2022 estimates, liver cancer ranked as the third leading cause of cancer-related death worldwide, with approximately 866, 000 new cases and 758, 000 deaths in 2022. The incidence and mortality of HCC remain nearly equal, largely due to inadequate early screening systems and the fact that most patients are diagnosed at intermediate or advanced stages (1). In China, the burden of HCC is particularly severe, with hepatitis B virus (HBV) infection being the leading cause, accounting for approximately 80% of HCC cases and driving the characteristic “HBV infection–cirrhosis–HCC” disease sequence (2, 3). The commonly used serum biomarker alpha-fetoprotein (AFP) has significant limitations: its sensitivity for detecting early-stage HCC (BCLC stage 0 or A) is less than 40% (4). Moreover, in the context of HBV-related active hepatitis and cirrhosis, liver cell damage and regeneration induced by viral replication can lead to elevated AFP levels in approximately 20% of patients with cirrhosis, compromising AFP’s ability to distinguish benign from malignant lesions (5). Consequently, more than half of HCC patients in China are diagnosed at intermediate to advanced stages (CNLC stage IIb–IIIa), resulting in a markedly reduced 5-year survival rate (6, 7). The complementary marker des-gamma-carboxy prothrombin (PIVKA-II, also known as DCP) also has notable limitations in diagnosing HBV-related HCC (8–10). A meta-analysis of DCP for HCC diagnosis reported an overall sensitivity of only 0. 66 (11) and in patients with HBV-related cirrhosis, serum DCP levels exhibit a false-positive rate of 15%–25%, with early-stage sensitivity of only approximately 35%–45% (11–14). Other biomarkers, such as α-L-fucosidase (AFU), have an early detection rate of less than 30% and are not directly associated with the pathogenesis of HBV-related HCC (15–17). Emerging markers, including circulating tumor cells (CTCs) and circulating tumor DNA (ctDNA), rely on complex techniques, are costly, lack specificity, and are not feasible for early screening as standalone tools (18, 19). Imaging modalities (e. g., ultrasound, CT, and MRI) offer advantages in tumor localization but conventional imaging still faces challenges in accurately characterizing subcentimeter nodules; novel diagnostic algorithms are therefore being developed to improve detection in this subgroup (20–22). In summary, there remains a lack of specific diagnostic biomarkers closely related to the pathogenesis of HBV-related HCC and capable of effectively identifying small, early-stage lesions. Therefore, this study was conducted to characterize ORC1 expression in HBV-related liver disease and to evaluate its association with HBV infection, disease progression, and prognosis.

Hepatitis B virus (HBV) integration-induced dysregulation of biological functions is a key mechanism underlying the development of HBV-related hepatocellular carcinoma (HCC) (23). In our preliminary study using clinical HBV-HCC samples to investigate viral integration sites, we observed frequent high-frequency insertion of the HBV S gene into regions adjacent to the ORC1 gene, suggesting that this genomic region is a preferred integration hotspot for HBV (23). The ORC1 gene is primarily involved in regulating the cell cycle and initiating DNA replication (24). Although genes such as TERT and MLL4 have been well established as common integration targets of HBV (25, 26), ORC1 has not yet been clearly defined as such. Importantly, ORC1 dysfunction directly interferes with the normal cell replication cycle, which differs from the mechanisms of previously identified integration target genes. Therefore, we hypothesize that HBV integration may contribute to ORC1 dysregulation, potentially leading to altered hepatocyte proliferation. This may contribute to the carcinogenic process of HBV-related HCC. Whether ORC1 can serve as a specific diagnostic biomarker for this disease requires further evaluation.

Building on the aforementioned preliminary findings and the functional characteristics of the ORC1 gene, this study aimed to characterize the expression profile of ORC1 in HBV-related liver diseases and to evaluate its association with HBV infection, disease progression, and prognosis. Using immunohistochemistry, we examined ORC1 expression in liver tissues with varying pathological states, and measured serum ORC1 in a small subset of patients with available samples. The diagnostic performance of tissue ORC1 was compared with that of conventional clinical tumor markers (AFP and DCP), and its prognostic value was assessed using TCGA-LIHC data.

## 2 Materials and methods

### 2.1 Study subjects

This study enrolled patients with pathologically confirmed normal liver tissue (adjacent to hepatic hemangioma), CHB, HBV-related liver cirrhosis (HBV-LC), and HBV-related HCC. Liver biopsy and surgically resected specimens were collected from the First Affiliated Hospital of Guangxi Medical University between July 2009 and August 2023 based on predefined inclusion and exclusion criteria. The Ethics Committee reviewed and approved the retrospective use of these archived specimens. The study protocol was approved by the Medical Ethics Committee of the First Affiliated Hospital of Guangxi Medical University (Approval No.: 2025-E0582, approved July 14, 2025), which confirmed that the study complied with medical ethics requirements. This was a retrospective study using archived pathological specimens and residual serum samples collected between 2009 and 2023. The Ethics Committee reviewed and approved the retrospective use of these archived specimens. All patient data were anonymized and de-identified prior to analysis.

### 2.2 Inclusion criteria

All participants had complete clinical and pathological documentation. Paraffin-embedded tissue blocks with well-preserved architecture were used for ORC1 detection by immunohistochemistry. All cases met the predefined diagnostic criteria for their respective groups. The HBV-negative control group comprised two distinct subgroups (hepatic hemangioma and non-HBV HCC), which were analyzed separately where relevant.

Patients in the CHB group were positive for HBsAg in both serum and liver tissue, with pathologically confirmed chronic hepatitis B.

Patients in the HBV- LC group were positive for HBsAg in both serum and liver tissue, with pathologically confirmed liver cirrhosis.

Patients in the HBV-related HCC group were positive for HBsAg in both serum and liver tissue, with pathologically confirmed primary HCC. These patients had received no prior radiotherapy, chemotherapy, endocrine therapy, targeted therapy, or other major treatments before surgery.

Patients in the normal liver tissue group (control) were negative for HBsAg and anti-HBc in serum, and negative for HBsAg and HBcAg in liver tissue (as confirmed by immunohistochemistry). Liver tissue was collected from the adjacent normal parenchyma of patients with pathologically confirmed hepatic hemangioma.

Patients in the non-HBV-associated HCC group were negative for HBsAg and anti-HBc in serum, and negative for HBsAg and HBcAg in liver tissue, with pathologically confirmed primary HCC. These patients had received no prior radiotherapy, chemotherapy, endocrine therapy, targeted therapy, or other major treatments before surgery.

### 2.3 Exclusion criteria

Patients were excluded if they met any of the following criteria: (1) receipt of preoperative radiotherapy, chemotherapy, or other treatments for HCC; (2) pathological diagnosis other than HCC; (3) incomplete clinical or pathological data; (4) liver failure or decompensated cirrhosis; or (5) age < 18 years.

### 2.4 Collection of tissue and serum specimens

All pathological sections required for this experiment were prepared from formalin-fixed, paraffin-embedded (FFPE) blocks that met the predefined inclusion criteria, as selected and verified by experienced pathologists. The blocks were labeled and sectioned at a thickness of 4–6 μm. The sections were then flattened and mounted using a section flotation bath, followed by baking in an oven at 80 °C for 1 hour. For HCC tissue blocks, sections with extensive tumor coverage, clear tissue architecture, and minimal hemorrhage or necrosis were preferentially selected. For adjacent non-cancerous or non-HCC cases, sections containing no visible cancer cells were chosen. Additionally, peripheral venous blood samples (5 mL each) were collected from 17 HBV-HCC patients with available preoperative serum prior to hepatectomy. No serum samples from chronic hepatitis B, HBV-related cirrhosis, or healthy controls were available for this analysis. The blood samples were centrifuged at 1, 500×g for 10 minutes at 4 °C, and the resulting serum was aliquoted and stored at −80 °C until batch analysis. All sample processing was performed in a blinded manner using a standardized protocol to ensure consistency and minimize batch effects.

### 2.5 Measurement of ORC1 in liver tissue

ORC1 protein expression in liver tissues was quantified by immunohistochemistry (IHC) and image analysis. After deparaffinization and antigen retrieval, sections were incubated with a primary antibody against ORC1 (catalog No. 13433-1-AP, Proteintech, Rosemont, IL, USA; dilution 1:200) overnight at 4 °C. Following incubation with a horseradish peroxidase (HRP)-conjugated secondary antibody (catalog No. SA00001-2, Proteintech, Rosemont, IL, USA; dilution 1:2000) for 1 hour at room temperature, sections were visualized using a diaminobenzidine (DAB) substrate kit (catalog No. ZLI-9018, ZSGB-BIO, Beijing, China) according to the manufacturer’s instructions. Integrated optical density (IOD) was used to represent relative ORC1 expression. For each section, five non-overlapping fields were captured at 40× magnification using an optical microscope. IOD values were measured with the Fiji-win64 image analysis system, and the mean IOD of the five fields was calculated as the relative ORC1 expression level for each case. IHC staining and IOD scoring were performed in a blinded manner, with the pathologist and the image analyst unaware of the clinical group assignments.

### 2.6 Measurement of serum ORC1

Serum ORC1 levels were measured using a commercial sandwich ELISA kit (Human ORC1 ELISA Kit, Cat. No.: ORC1-Hu-96T, Hong Kong, China; detection range: 156–10, 000 ng/L). All procedures were performed according to the manufacturer’s instructions, and each sample was measured in duplicate. Absorbance was measured at 450 nm, and ORC1 concentrations were calculated using a standard curve with R² ≥ 0. 99. Serum AFP and DCP levels were measured by the Department of Clinical Laboratory at the First Affiliated Hospital of Guangxi Medical University using clinically validated immunoassay platforms.

### 2.7 Public data acquisition

RNA-seq data and clinical information for HCC samples were obtained from the TCGA database (TCGA-LIHC cohort). The cohort was stratified into HBV-positive and HBV-negative groups based on clinical records and HBV molecular marker results, and normalized ORC1 expression data were extracted. The correlations between tissue ORC1 expression and DNA replication, G2M checkpoint, and tumor proliferation signatures, along with its cell-cycle dynamic expression profile, were sourced from the TCGA and CycleBase databases. ORC1 expression and overall survival in TCGA-LIHC were independently validated using GEPIA2 (http://gepia2.cancer-pku.cn/), with the following parameters: Dataset = LIHC; Group cutoff = Median; Methods = Overall Survival; Matched normal data = Match TCGA normal and GTEx data.

### 2.8 Statistical analysis

Statistical analyses were performed using SPSS and GraphPad Prism. Continuous data are presented as mean ± SD or median (IQR). Group comparisons were performed using the t-test, chi-square test (or Fisher’s exact test), and one-way ANOVA as appropriate. Spearman’s rank correlation was used for non-normally distributed data. Logistic regression was used to identify factors associated with high ORC1 expression, using the overall median IOD as the cutoff. ROC analysis with the Youden index was used to determine the optimal diagnostic cutoff and to evaluate the discrimination of ORC1, AFP, and their combination. Survival analysis was performed using the Kaplan-Meier method with log-rank tests, and TCGA-LIHC data were validated using GEPIA2. A two-tailed *P* < 0. 05 was considered statistically significant. Subgroup and correlation analyses were exploratory; formal correction for multiple comparisons was not applied. Model calibration was assessed using the Hosmer–Lemeshow goodness-of-fit test (multivariate logistic model: P = 0. 591; combined ORC1+AFP model: P = 0. 074 for HBV-HCC vs HBV-LC and P = 0. 964 for HBV-HCC vs CHB). Statistical analyses were performed using IBM SPSS Statistics version 27 and GraphPad Prism version 10. 1. 2.

### 2.9 Single-cell RNA-seq reanalysis

Single-cell RNA-seq data from GSE149614 were reanalyzed. Hepatocytes were identified based on the original cell-type annotations. ORC1 expression was compared between tumor and adjacent non-tumor hepatocytes, stratified by HBV status, using the Wilcoxon rank-sum test.

## 3 Results

### 3.1 General patient data

A total of 178 patients were included. The HBV-positive group consisted of chronic hepatitis B (CHB), HBV-related liver cirrhosis (HBV-LC), and HBV-related hepatocellular carcinoma (HBV-HCC) patients, whereas the HBV-negative group included hepatic hemangioma and non-HBV-related HCC (non-HBV-HCC) patients. Median transaminase levels in all groups were within clinical reference ranges (see **Table 1** for details). The HBV-negative control group comprised two distinct subgroups (hepatic hemangioma and non-HBV HCC), which were analyzed separately where relevant.

**Table 1:** Baseline characteristics of the study population.

Group	HBV positive (N=124)			HBV negative (N=54)	
	CHB group	HBV-LC group	HBV-HCC group	Hepatic hemangioma	Non-HBV-HCC group
Number	42	33	49	19	35
Male (%)	71.4	78.8	87.8	27.8	65.7
Age (years, mean ± SD),P>0.05	37.7± 9.4	41.7 ± 12.0	49.7 ± 9.7	45.3 ± 12.3	63.5 ± 10.1
ALT[U/L, M,(Q1-Q3)]	31.5(19,48.5)	33(25,56)	31(25,41)	15(10.5,23.2)	21(14,35.5)
AST[U/L, M,(Q1-Q3)]	29(22,37.5)	39(29.5,58)	31(25,41)	21(17,25.8)	28(20,33)
AFP (ng/ml, M[Q1,Q3])	2.1 [1.8, 3.3]	8.3 [3.8, 30.0]	13.5 [3.8, 340.6]	2.1 [1.6, 3.1]	46.6 [4.3, 1019.8]
DCP (mAu/mL, M[Q1,Q3])	-	-	74.1 [28.5, 460.6]	23.2 [17.5, 24.5]	305.4 [75.3, 1995.3]

Data are presented as mean ± SD or median (IQR). DCP was not routinely measured in the CHB and HBV-LC groups..

### 3.2 HBV infection upregulates ORC1 expression

Tissue ORC1 expression was associated with HBV infection, as shown by immunohistochemistry. The HBV-positive group (CHB, HBV-LC, and HBV-HCC) is presented in **Figures 1A, B, D**, and the HBV-negative group (hepatic hemangioma, n = 19; non-HBV-HCC, n = 35) in **Figures 1C, E**. ORC1 expression increased progressively from CHB to HBV-LC to HBV-HCC. The HBV-HCC group exhibited the highest expression level (0. 27 ± 0. 12), which was significantly higher than that in the HBV-LC group (0. 23 ± 0. 05, *P* < 0. 05) and the CHB group (0. 22 ± 0. 05, *P* < 0. 05). Moreover, all HBV-positive subgroups had significantly higher ORC1 levels than the HBV-negative subgroups (non-HBV-HCC: 0. 16 ± 0. 09; hepatic hemangioma: 0. 12 ± 0. 03; both *P* < 0. 05; **Figure 1F**). Direct comparison showed that tissue ORC1 expression was significantly higher in HBV-HCC (0. 27 ± 0. 12, n=49) than in non-HBV-HCC (0. 16 ± 0. 09, n=35; mean difference = 0. 116, 95% CI 0. 070–0. 162, *P* < 0. 001; Cohen’s d = 1. 10, 95% CI 0. 63–1. 56; **Figures 1E, F**). These findings were validated using TCGA data, which demonstrated significantly higher ORC1 expression in tumor tissues than in normal liver tissues (**Figure 1G**), and in HBV-associated HCC than in non-HBV-associated HCC (median expression values: 2. 62 vs. 2. 10, *P* < 0. 001; **Figure 1H**). Collectively, our results and public database analyses consistently suggest that tissue ORC1 expression is elevated in HBV-infected liver.

**Figure 1:**
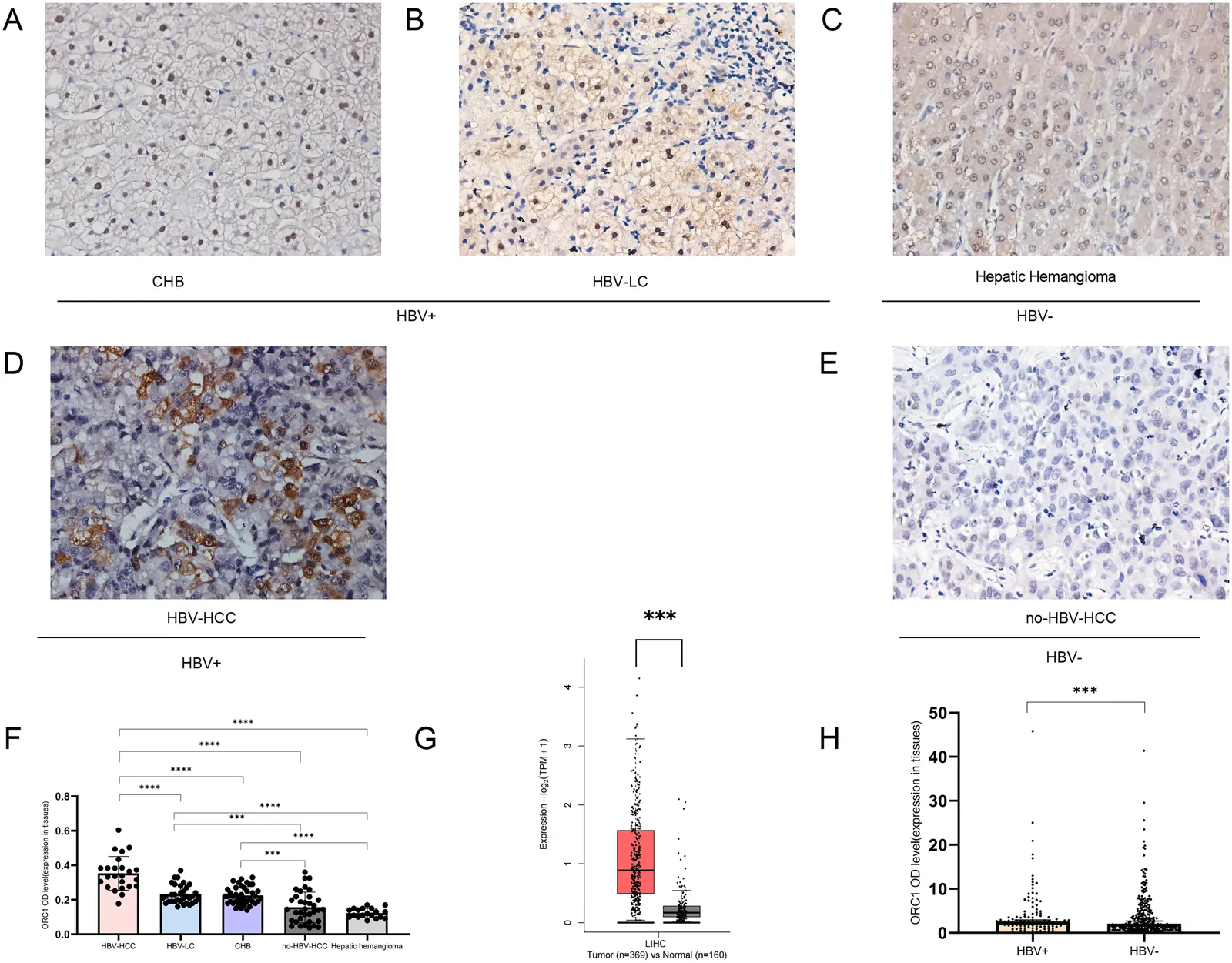
ORC1 expression in liver tissues. **(A)** Immunohistochemical staining of ORC1 in liver tissue from a patient with chronic hepatitis B (CHB) (40× magnification); **(B)** Immunohistochemical staining of ORC1 in liver tissue from a patient with HBV-related liver cirrhosis (HBV-LC) (40× magnification); **(C)** Immunohistochemical staining of ORC1 in liver tissue from a patient with hepatic hemangioma (40× magnification); **(D)** Immunohistochemical staining of ORC1 in tumor tissue from a patient with HBV-related hepatocellular carcinoma (HBV-HCC) (40× magnification); **(E)** Immunohistochemical staining of ORC1 in tumor tissue from a patient with non-HBV-HCC (40× magnification); **(F)** Comparison of tissue ORC1 expression levels between HBV-positive and HBV-negative groups in the study cohort;Direct comparison between HBV-HCC and non-HBV-HCC: mean difference = 0.116, 95% CI 0.070–0.162, P < 0.001; Cohen's d = 1.10. **(G)** ORC1 expression in TCGA-LIHC (GEPIA2): tumor tissues (n=369) vs normal liver tissues (n=160); **(H)** Comparison of ORC1 expression levels in tumor tissues between HBV-positive and HBV-negative groups in the TCGA hepatocellular carcinoma dataset.

ORC1 expression levels (mean IOD values) from the 178 patients were stratified using the overall median (0. 12) as the cutoff; values above 0. 12 were defined as high expression. Univariate logistic regression was performed with high ORC1 expression as the outcome and the following factors as covariates: age, sex, HBV infection (infected vs. non-infected), ALT, AST, AFP, DCP, TBIL, ALB, PT, and INR. In the univariate analysis, HBV infection, sex, and age were identified as factors significantly associated with high ORC1 expression. These three variables were then entered into a multivariate logistic regression model, which confirmed HBV infection as an independent factor associated with high ORC1 expression (adjusted OR = 11. 91, 95% CI 4. 02–35. 32, P<0. 001). Importantly, this association remained significant after additional adjustment for HCC status (adjusted OR = 11. 91, *P*<0. 001; HCC status: OR = 0. 73, *P* = 0. 573), indicating that it is not attributable to HCC status. The overall likelihood ratio test of the model was significant (χ² = 32. 45, df = 3, *P* < 0. 001), demonstrating substantial joint explanatory power of the included independent variables for the outcome (Univariate results shown in **Table 2**, multivariate results in **Table 3**). Correlation analysis showed that neither tissue ORC1 (n=124; Pearson R = 0. 067, 95% CI −0. 111–0. 240, *P* = 0. 460) nor serum ORC1 (n=17; Pearson R = −0. 081, 95% CI −0. 540–0. 416, *P* = 0. 758) was significantly correlated with serum HBV DNA load. External validation using GSE14520/GPL571 confirmed that ORC1 was significantly upregulated in tumor tissues compared with non-tumor tissues (logFC = 0. 228, *P* = 1. 22×10^–3^, adjusted *P* = 5. 10×10^–3^). In GSE14520, ORC1 expression did not differ significantly between chronic-carrier tumors (CC, n=156) and active-replication tumors (AVR-CC, n=56) (mean difference +0. 01 on the log2 scale; Mann–Whitney *P* = 0. 78; Cohen’s d = 0. 03), supporting that ORC1 upregulation is associated with HBV infection rather than with active viral replication.

**Table 2:** Univariate logistic regression analysis of factors associated with high ORC1 expression.

Variable	Gender	Age	HBV positivity	DCP	ALT	AST	TB	ALB	INR	PT
Coding / Variable type	Male=1, Female=0	Continuous variable	Positive=1 Negative=0	Continuous variable	Continuous variable	Continuous variable	Continuous variable	Continuous variable	Continuous variable	Continuous variable
OR	2.40	0.96	11.12	1.00	0.99	0.99	1.02	1.05	3.88	0.98
95% CI	1.09-5.30	0.94-1.00	4.52-27.32	1.00-1.00	0.99-1.00	0.99-1.00	0.98-1.06	0.96-1.15	0.06-254.24	0.75-1.29
*P*	0.03	0.02	<0.001	0.19	0.54	0.19	0.46	0.31	0.53	0.89

**Table 3:** Multivariate logistic regression analysis.

Variable	B	SE	Wald	OR	95% CI	*P*
HBV infection	2.478	0.555	19.961	11.91	4.02–35.32	<0.001
HCC status	−0.322	0.572	0.317	0.73	0.24–2.22	0.573
Age	0.012	0.021	0.310	1.01	0.97–1.05	0.578
Gender	0.040	0.491	0.007	1.04	0.40–2.73	0.935

Variables with *P* < 0.05 in univariate analysis were included in the multivariate model.

### 3.3 Tissue-specific expression of ORC1 in HBV-related hepatocellular carcinoma

ORC1 expression was elevated in both HBV-HCC and non-HBV-HCC tumor tissues compared with matched adjacent non-tumor tissues (HBV-HCC: 0. 272 vs 0. 115, n=49, *P*<0. 001; non-HBV-HCC: 0. 156 vs 0. 091, n=35, *P*<0. 001), but the magnitude of upregulation was significantly greater in HBV-HCC than in non-HBV-HCC (0. 272 vs 0. 156, *P*<0. 001; Cohen’s d=1. 10). ORC1 expression was also strongly correlated with DNA replication (R = 0. 82), G2/M checkpoint (R = 0. 88), and tumor proliferation signatures (R = 0. 85) (all *P*<0. 001; **Figures 2A–C**). High ORC1 expression was associated with poorer prognosis (HR = 1. 7, *P* = 0. 0026; **Figure 2D**). Tissue and serum ORC1 levels were moderately correlated in the 17 HBV-HCC patients with available serum (Spearman rs=0. 554, 95% CI 0. 085–0. 822, *P* = 0. 021; **Figure 2E**), but not in adjacent non-tumor tissues or in non-HBV-HCC patients (all *P*>0. 05; **Figures 2F–H**). Serum ORC1 concentrations in the 17 HBV-HCC patients with available serum were 1479. 56 ± 594. 61 ng/L (mean ± SD), with a median of 1481. 13 ng/L (IQR 1004. 21–1912. 20 ng/L). These findings suggest that serum ORC1 may be partly tumor-derived, though validation in larger cohorts is required.

**Figure 2:**
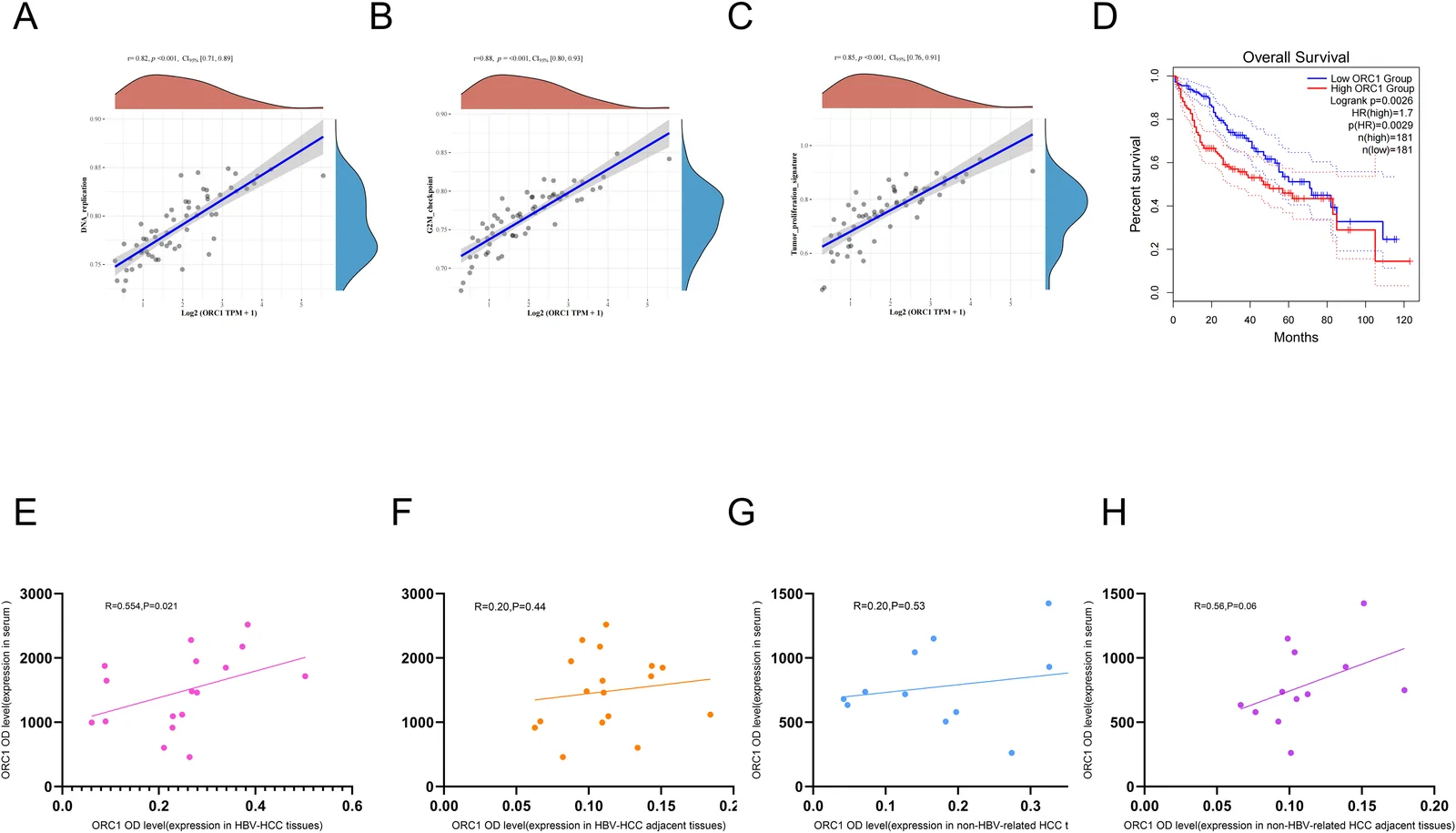
Correlations of ORC1 expression with signaling pathways and survival outcomes. **(A)** Correlation of ORC1 expression in tumor tissues with the DNA replication pathway; **(B)** Correlation of ORC1 expression with the G2/M checkpoint pathway; **(C)** Correlation of ORC1 expression with the tumor proliferation signature; **(D)** Overall survival curves for HBV-HCC patients stratified by high vs. low ORC1 expression (median cutoff) in TCGA-LIHC; HR=1.7, log-rank P=0.0026 (n=181 per group). Data were obtained from GEPIA2; **(E)** Correlation between serum ORC1 levels and ORC1 expression in HBV-HCC tumor tissues (Spearman rs=0.554, 95% CI 0.085–0.822, P=0.021, n=17); **(F)** Correlation between serum ORC1 levels and ORC1 expression in adjacent non-tumor tissues of HBV-HCC patients; **(G)** Correlation between serum ORC1 levels and ORC1 expression in tumor tissues of non-HBV-HCC patients; **(H)** Correlation between serum ORC1 levels and ORC1 expression in adjacent non-tumor tissues of non-HBV-HCC patients.

### 3.4 Missed diagnosis rates of ORC1, AFP, and DCP for HBV-HCC

A total of 49 patients with HBV-related hepatocellular carcinoma (HBV-HCC) were enrolled in this study, with clinicopathologically confirmed HCC serving as the diagnostic gold standard. The predefined positive diagnostic thresholds were set as follows: serum AFP ≥ 400 ng/mL, serum DCP ≥ 40 mAu/mL, and tissue ORC1 IOD > 0. 22 (ROC-derived cutoff). The missed diagnosis rates were 73. 47% (36/49) for AFP, 26. 53% (13/49) for DCP, and 22. 45% (11/49) for ORC1. To address the threshold-dependence of AFP performance, the missed diagnosis rate was also calculated using the conventional screening cutoff of 20 ng/mL, which was 51. 02% (25/49). The ORC1 missed diagnosis rate was significantly lower than that of AFP at both thresholds (*P* < 0. 001 for AFP ≥ 400 ng/mL; *P* = 0. 004 for AFP ≥ 20 ng/mL, McNemar test).

### 3.5 Tissue ORC1 diagnosis for HBV-HCC is unaffected by serum AFP and DCP

Using predefined thresholds for serum AFP and DCP, 49 HBV-HCC patients were divided into positive and negative groups. Tissue ORC1 expression showed no significant difference between AFP-positive and AFP-negative groups (0. 33 vs 0. 25, *P* > 0. 05; **Figure 3A**) or between DCP-positive and DCP-negative groups (0. 25 vs 0. 28, *P* > 0. 05; **Figure 3B**). Among all patients, the Venn diagrams showed that tissue ORC1 positivity exceeded that of DCP when AFP was positive (**Figure 3C**), and was comparable to DCP when AFP was negative (**Figure 3D**). Regardless of DCP status, tissue ORC1 positivity rate was consistently higher than that of AFP (**Figures 3E, F**). In the clinically relevant comparisons, tissue ORC1 showed moderate discrimination between HBV-HCC and HBV-LC (AUC = 0. 64, 95% CI 0. 52–0. 76) and between HBV-HCC and CHB (AUC = 0. 66, 95% CI 0. 55–0. 78). AFP alone achieved higher AUCs than ORC1 alone in both comparisons (0. 66 vs 0. 64 for HBV-HCC vs HBV-LC; 0. 86 vs 0. 66 for HBV-HCC vs HBV-CHB; **Figures 3G, H**). At the 0. 22 cutoff, its specificity was 54. 55% (vs HBV-LC) and 51. 22% (vs CHB; **Figures 3G, H**). A combined ORC1+AFP model yielded AUCs of 0. 67 (vs HBV-LC) and 0. 86 (vs HBV-CHB; **Figures 3G, H**). Adding age further improved diagnostic performance: the ORC1+AFP+Age model achieved AUCs of 0. 77 (95% CI 0. 66–0. 87) for HBV-HCC vs HBV-LC and 0. 93 (95% CI 0. 87–0. 98) for HBV-HCC vs HBV-CHB (**Table 4**).

**Figure 3:**
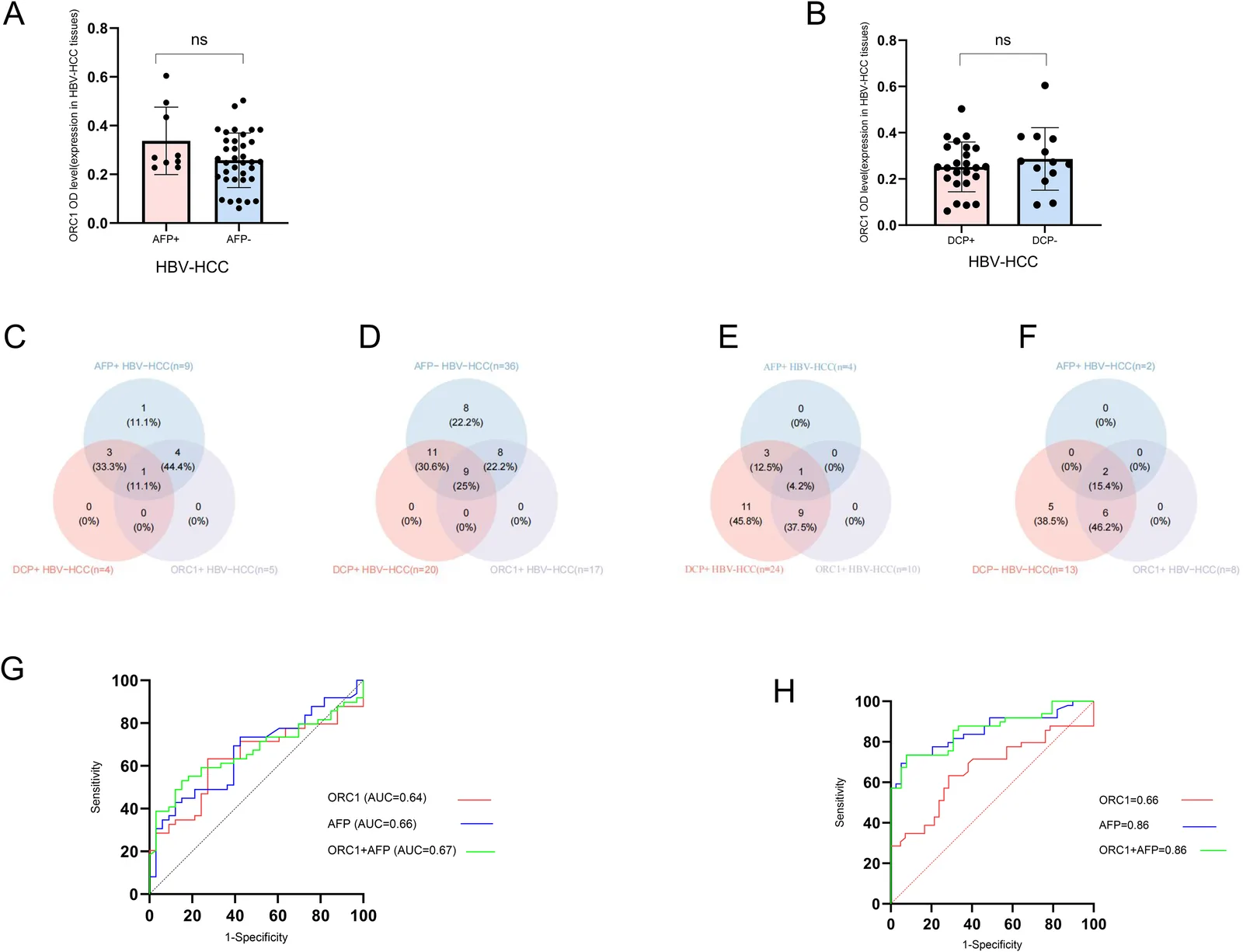
Diagnostic performance of tissue ORC1 in HBV-HCC. **(A)** Tissue ORC1 expression: AFP-positive vs. AFP-negative; **(B)** Tissue ORC1 expression: DCP-positive vs. DCP-negative; **(C)** Venn diagram showing the overlap of ORC1, AFP, and DCP positivity in AFP-positive patients; **(D)** Venn diagram showing the overlap of ORC1, AFP, and DCP positivity in AFP-negative patients; **(E)** Venn diagram showing the overlap of ORC1, AFP, and DCP positivity in DCP-positive patients; **(F)** Venn diagram showing the overlap of ORC1, AFP, and DCP positivity in DCP-negative patients; **(G)** ROC curves of tissue ORC1, AFP, and the combined ORC1+AFP model for discriminating HBV-HCC from HBV-LC (AUCs: ORC1=0.64, AFP=0.66, ORC1+AFP=0.67); **(H)** ROC curves of tissue ORC1, AFP, and the combined ORC1+AFP model for discriminating HBV-HCC from CHB (AUCs: ORC1=0.66, AFP=0.86, ORC1+AFP=0.86).

**Table 4:** Diagnostic performance of tissue ORC1 and combined models (ORC1+AFP, ORC1+AFP+Age) for HBV-HCC.

Comparison	AUC (95% CI)	Sensitivity	Specificity	PPV	NPV
HBV-HCC vs HBV-LC (ORC1)	0.64 (0.52–0.76)	77.55%	54.55%	71.70%	62.07%
HBV-HCC vs CHB (ORC1)	0.66 (0.55–0.78)	77.55%	51.22%	65.52%	65.63%
HBV-HCC vs HBV-LC (ORC1+AFP)	0.67(0.55-0.78)	–	–	–	–
HBV-HCC vs CHB (ORC1+AFP)	0.86(0.78-0.94)	–	–	–	–
HBV-HCC vs HBV-LC (ORC1+AFP+Age)	0.77 (0.66–0.87)	–	–	–	–
HBV-HCC vs CHB (ORC1+AFP+Age)	0.93 (0.87–0.98)	–	–	–	–

Diagnostic performance of tissue ORC1 and combined models for distinguishing HBV-HCC from HBV-LC and CHB. AUC, area under the receiver operating characteristic curve; PPV, positive predictive value; NPV, negative predictive value. For the combined models, sensitivity, specificity, PPV, and NPV are not shown because the logistic model generates only a continuous predicted probability; AUC is the primary performance metric.

### 3.6 Tissue ORC1 expression across tumor diameter subgroups

Among 49 HBV-HCC patients stratified by maximum tumor diameter <1 cm (n=5), 1–<2 cm (n=9), and ≥2 cm (n=35), tissue ORC1 expression did not differ significantly across groups (0. 25 vs 0. 24 vs 0. 26, *P* > 0. 05; **Figure 4A**). In contrast, serum log10(AFP) levels differed significantly among the three groups (0. 98 vs 0. 88 vs 1. 58; *P* < 0. 05; **Figure 4B**), and serum log10(DCP) levels differed significantly between the <1 cm and ≥2 cm groups (1. 52 vs 1. 98; *P* < 0. 05; **Figure 4C**).

**Figure 4:**
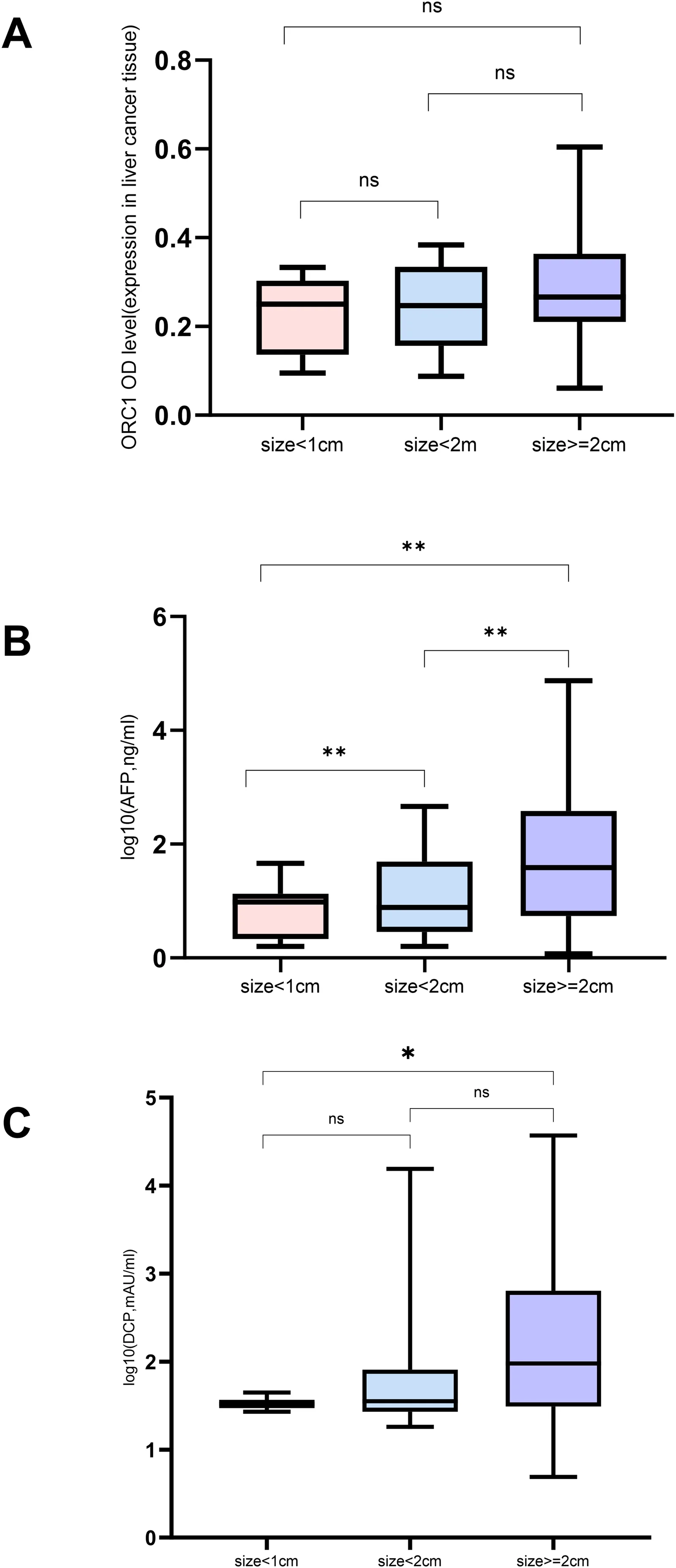
Tissue ORC1, serum AFP, and serum DCP levels stratified by tumor diameter in HBV-HCC. **(A)** Tissue ORC1 expression stratified by maximum tumor diameter (<1 cm, 1–<2 cm, ≥2 cm); **(B)** Serum log10(AFP) stratified by tumor diameter; **(C)** Serum log10(DCP) stratified by tumor diameter. Data are presented as median (IQR). Statistical significance was determined by one-way ANOVA followed by post-hoc tests. *P<0.05, **P<0.01; ns, not significant.

### 3.7 Single-cell RNA-seq analysis of ORC1 expression in hepatocytes

In the GSE149614 single-cell dataset, ORC1 was significantly higher in HBV-HCC tumor hepatocytes than in matched adjacent normal hepatocytes (0. 034 vs 0. 002, *P* = 9. 0e-11; **Figure 5**). Importantly, HBV-HCC tumor hepatocytes showed significantly higher ORC1 than non-HBV (HCV-positive or etiology-negative) tumor hepatocytes (0. 034 vs 0. 007, *P*<2. 2e-16; **Figure 5**). This analysis is based on 10 patients (5 HBV-positive, 2 HCV-positive, and 3 etiology-negative).

**Figure 5:**
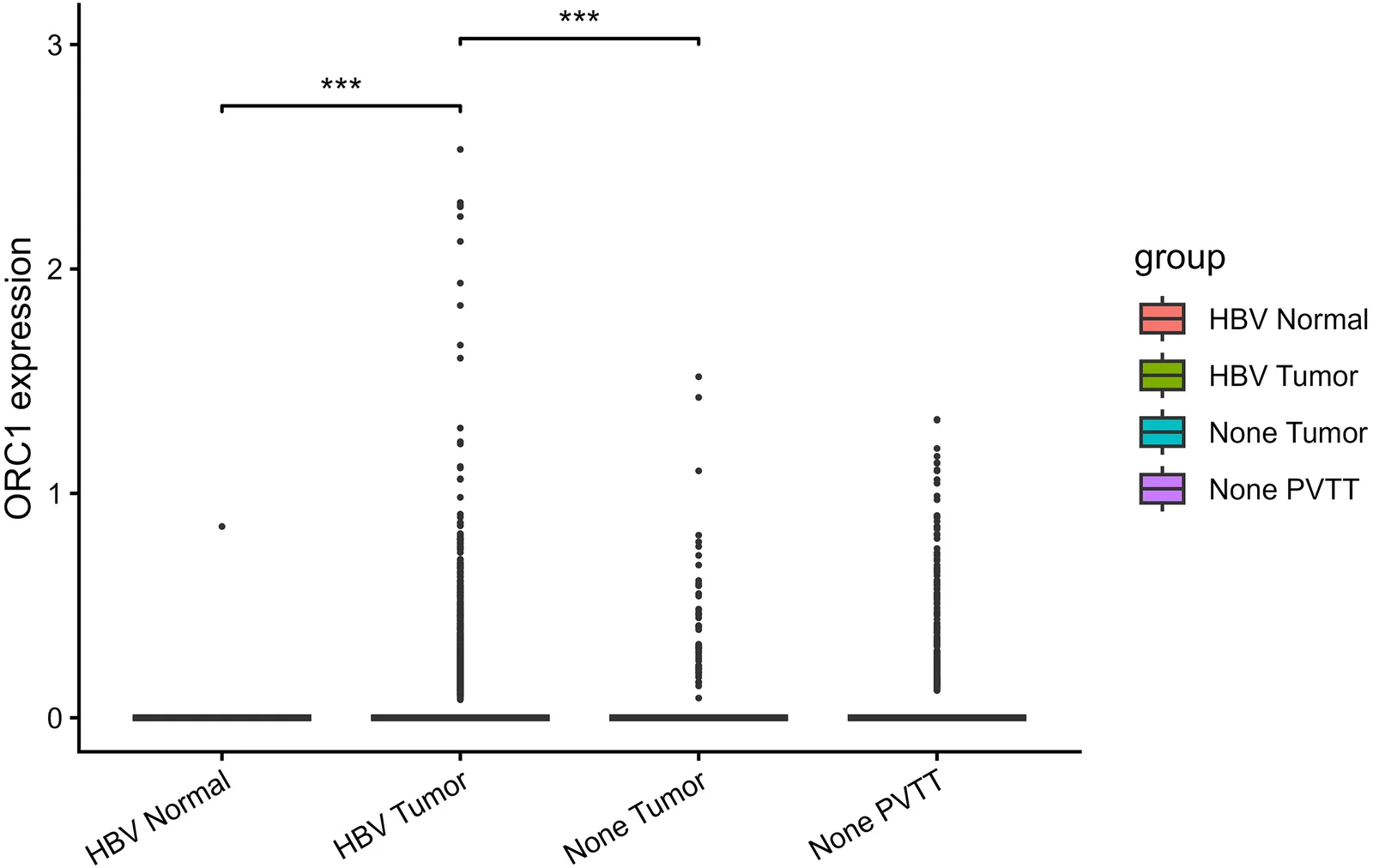
Single-cell RNA-seq analysis of ORC1 expression in hepatocytes from GSE149614. ORC1 expression was compared between HBV-HCC tumor hepatocytes (n=5121), HBV-HCC adjacent normal hepatocytes (n=487), non-HBV (HCV-positive or etiology-negative) tumor hepatocytes (n=3153), and non-HBV (HCV-positive or etiology-negative) PVTT hepatocytes (n=3329). This analysis is based on 10 patients (5 HBV-positive, 2 HCV-positive, and 3 etiology-negative). Wilcoxon rank-sum test: HBV Tumor vs HBV Normal, P=9.0e-11; HBV Tumor vs None Tumor, P<2.2e-16; None Tumor vs None PVTT, P<2.2e-16. PVTT, portal vein tumor thrombus.

## 4 Discussion

In China, over 80% of primary hepatocellular carcinoma (HCC) cases arise secondary to hepatitis B virus (HBV) infection. The disease typically follows a progressive course from chronic hepatitis to cirrhosis and ultimately to HCC, during which early diagnosis remains challenging. Current conventional tumor markers, such as AFP and DCP, are susceptible to interference from liver fibrosis and hepatic inflammation, resulting in suboptimal sensitivity and specificity, particularly for early-stage HCC. Consequently, biomarkers directly linked to HBV-driven carcinogenesis may offer improved diagnostic accuracy. Given that the HBV genome tends to integrate into the host origin recognition complex subunit 1 (ORC1) locus, we examined whether ORC1 expression is altered in HBV-related liver disease and how it relates to disease progression and clinical outcomes.

ORC1 is a core regulator of DNA replication, cell cycle progression, and genomic stability, mediating replication licensing to ensure orderly DNA replication under physiological conditions. HBV integration may contribute to ORC1 overexpression, which could potentially disrupt replication homeostasis, induce replication stress, and interfere with the RB-E2F pathway, potentially leading to escape from G1/S checkpoint control (**Figure 6**; cell-cycle dysregulation data from CycleBase), and thereby contributing to genomic instability, chromosomal aberrations, and malignant proliferation in HBV-HCC (27). Our previous finding of frequent ORC1-HBV integration events suggested a functional role for ORC1 during HBV infection (23), which is supported by the present study showing markedly upregulated tissue ORC1 expression in HBV-infected individuals, with broader and more intense ORC1 staining in HBV-positive versus negative hepatocytes (**Figures 1A–E**); logistic regression identified HBV positivity as an independent factor associated with high ORC1 expression (**Tables 2**, **3**). High ORC1 expression was consistently observed across HBV-infected hepatitis, cirrhosis, and HCC tissues (**Figure 1F**), with clear differences between HBV-HCC and non-HBV-HCC tissues (**Figure 1H**) and external validation (**Figure 1G**). A direct comparison further confirmed that tissue ORC1 was significantly higher in HBV-HCC than in non-HBV-HCC, supporting a quantitative association between HBV infection and ORC1 upregulation. Stepwise increases in ORC1 expression were observed across disease progression (**Figure 1F**); however, no significant correlation was found between ORC1 expression and serum HBV DNA load, suggesting that ORC1 upregulation is associated with HBV infection rather than with active viral replication. Consistent with this, ORC1 expression did not differ between chronic-carrier and active-replication tumors in GSE14520. A plausible mechanistic interpretation is that ORC1 is a cell-cycle/E2F-responsive gene reflecting proliferative state, whereas serum HBV DNA reflects contemporaneous viral production measured at a single time point; this temporal and compartmental mismatch may explain the lack of correlation. These data do not, however, establish the integration mechanism. Based on a single, uncorroborated prior observation from our previous study (23) and ORC1’s role as a core cell cycle regulator (28), HBV integration near the ORC1 locus may represent one possible mechanism contributing to ORC1 dysregulation, potentially leading to sustained proliferative stress and carcinogenic risk (29). Further functional studies are needed to confirm this hypothesis. These findings suggest an association between aberrant ORC1 expression and HBV-related hepatocarcinogenesis. Importantly, the association between HBV infection and ORC1 upregulation remained significant after adjusting for HCC status, indicating that this association is not merely a consequence of HCC. External validation using an independent GEO cohort (GSE14520/GPL571) further confirmed that ORC1 was significantly upregulated in tumor tissues compared with non-tumor tissues. Consistent with these findings, single-cell RNA-seq analysis of GSE149614 showed that ORC1 was significantly higher in HBV-HCC tumor hepatocytes than in non-HBV (HCV-positive or etiology-negative) tumor hepatocytes, supporting a quantitative association between HBV infection and ORC1 upregulation.

**Figure 6:**
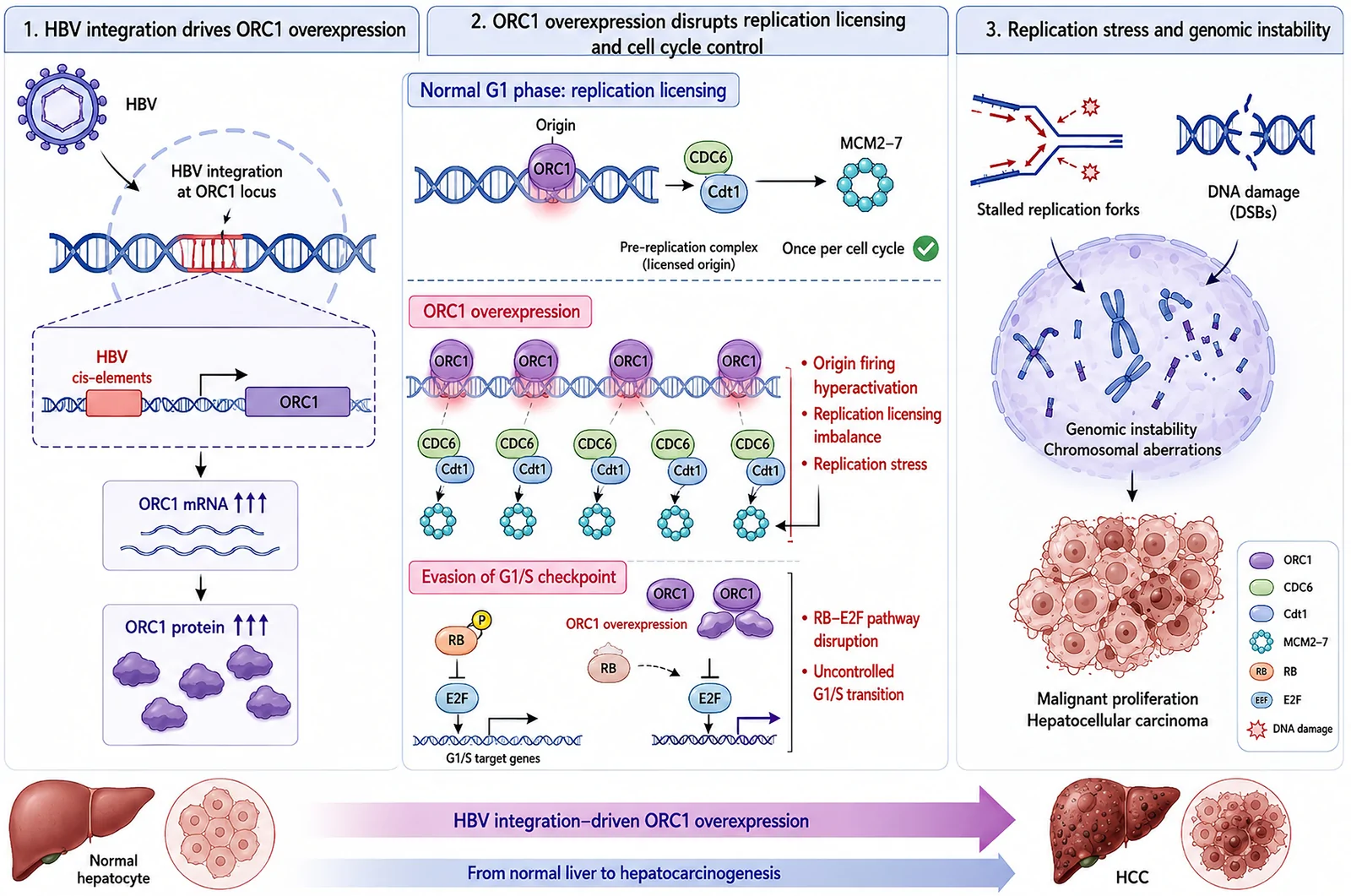
Proposed model linking HBV integration and ORC1 dysregulation to HBV-HCC progression. HBV integration into the ORC1 locus may drive ORC1 overexpression, which could disrupt replication licensing and cell cycle control, leading to replication stress, genomic instability, and malignant proliferation in HBV-HCC.

ORC1 is primarily involved in DNA replication initiation, cell cycle regulation, and the replication stress response pathway (**Figures 2A–C**). Analysis of TCGA transcriptomic and clinical data for HBV-HCC showed that high ORC1 expression was significantly associated with shorter overall survival compared with low expression (**Figure 2D**), indicating its potential value for risk stratification. Beyond its prognostic value, we further investigated the origin of circulating ORC1. Based on tissue expression data, our correlation analysis demonstrated that serum ORC1 levels were moderately correlated with tumor ORC1 expression in a small subset of HBV-HCC patients (**Figure 2E**), with no significant association observed in adjacent non-tumor tissues or non-HBV-related HCC tissues (**Figures 2F–H**). These findings suggest that circulating ORC1 may be partly tumor-derived.

The missed diagnosis rate of tissue ORC1 for HBV-HCC (22. 45%) was lower than that of AFP at both the guideline-recommended threshold (73. 47% at ≥400 ng/mL) and the conventional screening threshold (51. 02% at ≥20 ng/mL), and comparable to that of DCP (26. 53%). Notably, ORC1 remained effective in detecting HBV-HCC among AFP-negative patients, suggesting potential complementary value. Tissue ORC1 expression was unaffected by AFP or DCP status (**Figures 3A, B**). As shown in the Venn diagrams, ORC1 maintained an independent positive distribution pattern regardless of AFP or DCP positivity (**Figures 3C–F**). The combined ORC1+AFP model showed superior diagnostic performance in both comparisons, and integrating age further improved diagnostic accuracy, supporting the value of combining ORC1 with clinical variables. Although AFP alone achieved higher AUCs than ORC1 alone, the combination of ORC1 and AFP (and further with age) improved diagnostic performance, supporting ORC1 as a complementary rather than standalone marker. We further examined the association between ORC1 expression and tumor diameter. Tissue ORC1 expression did not fluctuate significantly with tumor diameter (**Figure 4A**). In contrast, for extremely early-stage small HCC (<1 cm; **Figure 4B**), both AFP and DCP levels showed significant differences or trending changes (**Figure 4C**). Notably, tissue ORC1 expression did not differ significantly across tumor diameter subgroups, indicating that ORC1 expression is independent of tumor size and may therefore be of potential value for detecting small HCC.

This study has several limitations. The serum ORC1 subset (n=17) and the tumor-diameter subgroups (<1 cm: n=5; 1–<2 cm: n=9; ≥2 cm: n=35) were limited by sample availability; these small sample sizes reduce statistical precision, and the corresponding findings should be interpreted as exploratory. (1) As a single-center retrospective analysis, the findings require validation in multicenter prospective cohorts with larger sample sizes. (2) Subgroup analyses were exploratory, and formal correction for multiple comparisons was not applied. (3) Although HBV DNA load was available in a subset of patients, tissue and serum ORC1 were not significantly correlated with HBV DNA load, and stratification by HBeAg status and antiviral treatment was not possible due to incomplete data. (4) Serum ORC1 was measured only in 17 HBV-HCC patients without non-HCC HBV controls; the tissue–serum correlation (n=17) was therefore unstable, and the diagnostic specificity of serum ORC1 could not be evaluated. (5) Cirrhosis status was collinear with the diagnostic groups (all HBV-LC patients had cirrhosis; all CHB patients did not), so a comprehensive nomogram integrating cirrhosis status could not be constructed. (6) The ORC1-HBV integration mechanism was derived from our previous study and has not been independently replicated or functionally validated; in that prior cohort, standalone serum ORC1 showed modest performance (AUC 0. 59). Single-cell validation was performed using the publicly available GSE149614 dataset, which confirmed greater ORC1 upregulation in HBV-HCC tumor hepatocytes than in non-HBV-HCC tumor hepatocytes.

## 5 Conclusions

ORC1 is upregulated in HBV-infected liver and its expression rises along the CHB–cirrhosis–HCC sequence. Tissue ORC1 displayed moderate diagnostic performance for HBV-HCC, supporting its role as an adjunct to existing markers. Its expression was independent of tumor diameter, a feature that may aid in identifying small HCC. High ORC1 expression correlated with worse overall survival in TCGA-LIHC. Collectively, ORC1 appears to function mainly as a proliferation- and progression-associated marker in HBV-related liver disease, with its primary clinical relevance in prognostication.

## Data Availability

The original data are available from the corresponding author on reasonable request. Public datasets analyzed in this study are available from TCGA (https://www.cancer.gov/tcga) and GEO (GSE149614, GSE14520).
